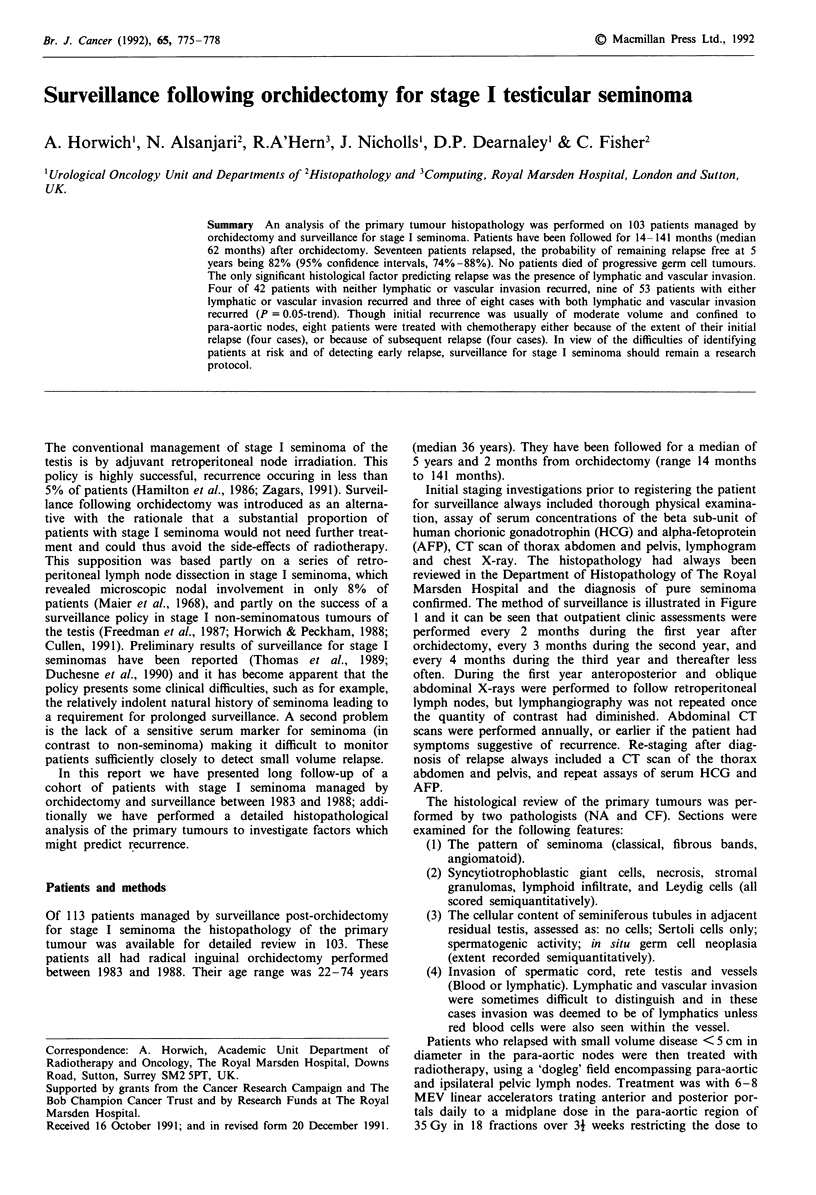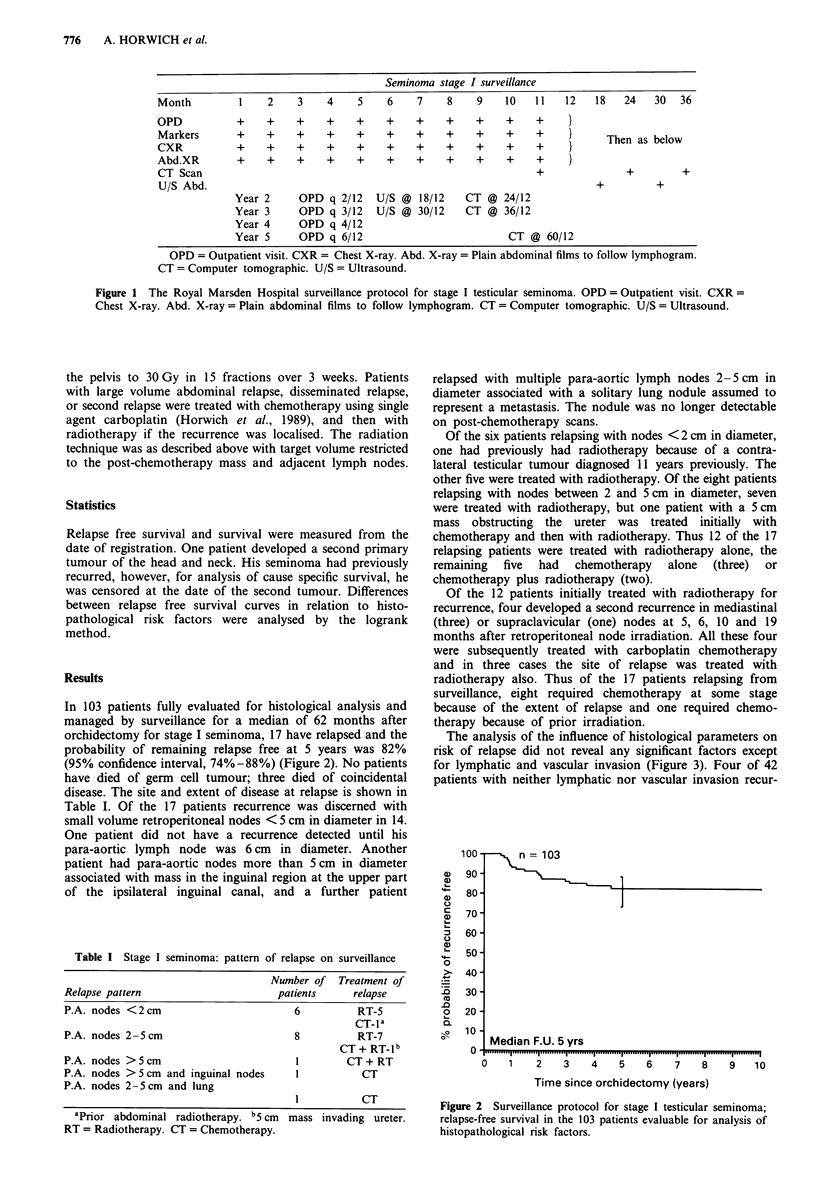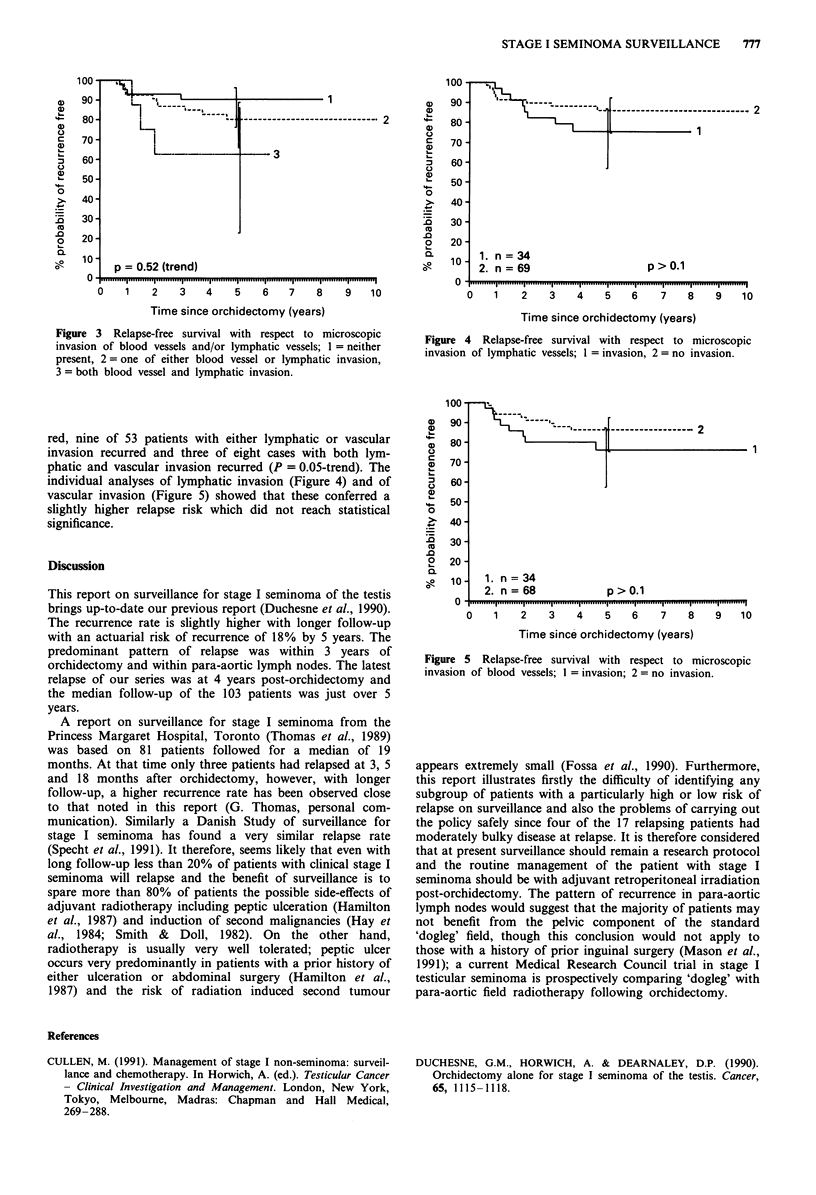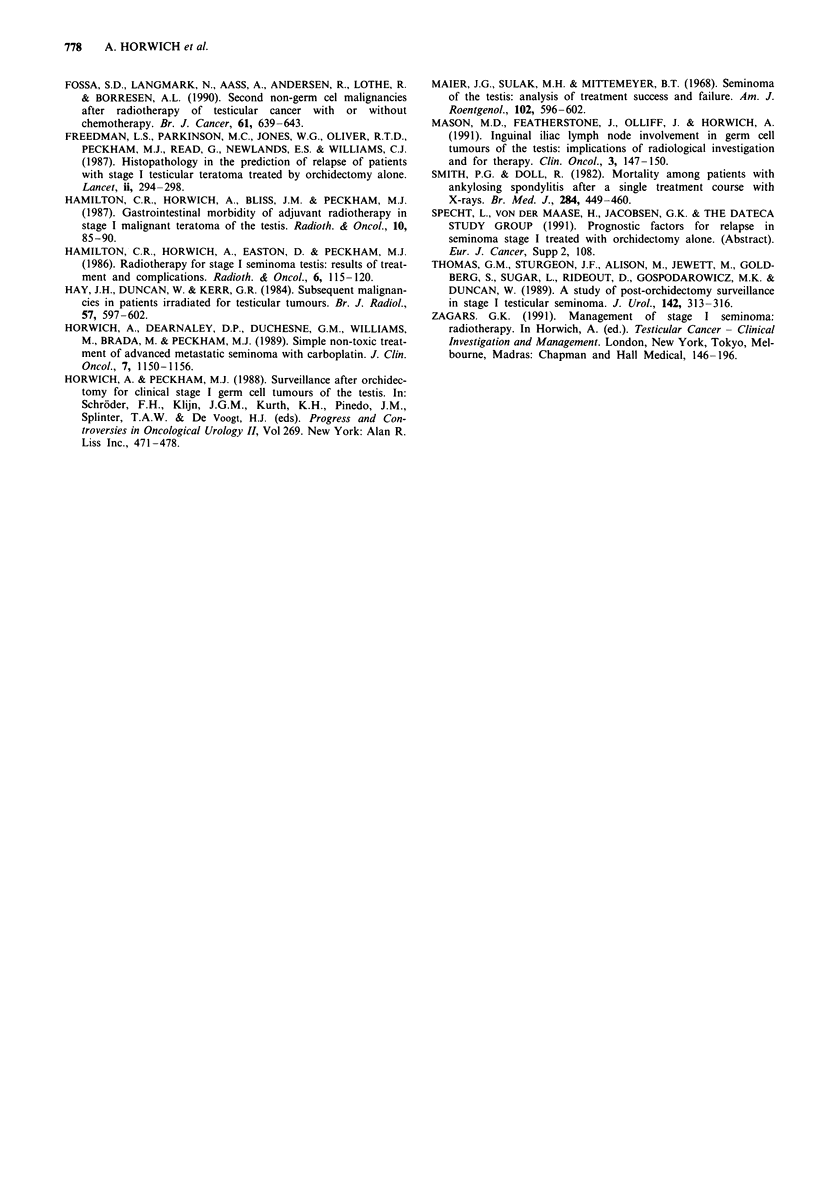# Surveillance following orchidectomy for stage I testicular seminoma.

**DOI:** 10.1038/bjc.1992.164

**Published:** 1992-05

**Authors:** A. Horwich, N. Alsanjari, R. A'Hern, J. Nicholls, D. P. Dearnaley, C. Fisher

**Affiliations:** Urological Oncology Unit, Royal Marsden Hospital, London, UK.

## Abstract

An analysis of the primary tumour histopathology was performed on 103 patients managed by orchidectomy and surveillance for stage I seminoma. Patients have been followed for 14-141 months (median 62 months) after orchidectomy. Seventeen patients relapsed, the probability of remaining relapse free at 5 years being 82% (95% confidence intervals, 74%-88%). No patients died of progressive germ cell tumours. The only significant histological factor predicting relapse was the presence of lymphatic and vascular invasion. Four of 42 patients with neither lymphatic or vascular invasion recurred, nine of 53 patients with either lymphatic or vascular invasion recurred and three of eight cases with both lymphatic and vascular invasion recurred (P = 0.05-trend). Though initial recurrence was usually of moderate volume and confined to para-aortic nodes, eight patients were treated with chemotherapy either because of the extent of their initial relapse (four cases), or because of subsequent relapse (four cases). In view of the difficulties of identifying patients at risk and of detecting early relapse, surveillance for stage I seminoma should remain a research protocol.


*Br. J. Cancer* (1992), **65**, 775–778 © Macmillan Press Ltd., 1992

### Surveillance following orchidectomy for stage I testicular seminoma

A. Horwich[1], N. Alsanjari[2], R.A'Hern[3], J. Nicholls[1], D.P. Dearnaley[1] & C. Fisher[2]

[1]*Urological Oncology Unit and Departments of* [2]*Histopathology and* [3]*Computing, Royal Marsden Hospital, London and Sutton, UK.*

**Summary** An analysis of the primary tumour histopathology was performed on 103 patients managed by orchidectomy and surveillance for stage I seminoma. Patients have been followed for 14–141 months (median 62 months) after orchidectomy. Seventeen patients relapsed, the probability of remaining relapse free at 5 years being 82% (95% confidence intervals, 74%–88%). No patients died of progressive germ cell tumours. The only significant histological factor predicting relapse was the presence of lymphatic and vascular invasion. Four of 42 patients with neither lymphatic or vascular invasion recurred, nine of 53 patients with either lymphatic or vascular invasion recurred and three of eight cases with both lymphatic and vascular invasion recurred ($P = 0.05$-trend). Though initial recurrence was usually of moderate volume and confined to para-aortic nodes, eight patients were treated with chemotherapy either because of the extent of their initial relapse (four cases), or because of subsequent relapse (four cases). In view of the difficulties of identifying patients at risk and of detecting early relapse, surveillance for stage I seminoma should remain a research protocol.

The conventional management of stage I seminoma of the testis is by adjuvant retroperitoneal node irradiation. This policy is highly successful, recurrence occuring in less than 5% of patients (Hamilton *et al.*, 1986; Zagars, 1991). Surveillance following orchidectomy was introduced as an alternative with the rationale that a substantial proportion of patients with stage I seminoma would not need further treatment and could thus avoid the side-effects of radiotherapy. This supposition was based partly on a series of retroperitoneal lymph node dissection in stage I seminoma, which revealed microscopic nodal involvement in only 8% of patients (Maier *et al.*, 1968), and partly on the success of a surveillance policy in stage I non-seminomatous tumours of the testis (Freedman *et al.*, 1987; Horwich & Peckham, 1988; Cullen, 1991). Preliminary results of surveillance for stage I seminomas have been reported (Thomas *et al.*, 1989; Duchesne *et al.*, 1990) and it has become apparent that the policy presents some clinical difficulties, such as for example, the relatively indolent natural history of seminoma leading to a requirement for prolonged surveillance. A second problem is the lack of a sensitive serum marker for seminoma (in contrast to non-seminoma) making it difficult to monitor patients sufficiently closely to detect small volume relapse.

In this report we have presented long follow-up of a cohort of patients with stage I seminoma managed by orchidectomy and surveillance between 1983 and 1988; additionally we have performed a detailed histopathological analysis of the primary tumours to investigate factors which might predict recurrence.

### Patients and methods

Of 113 patients managed by surveillance post-orchidectomy for stage I seminoma the histopathology of the primary tumour was available for detailed review in 103. These patients all had radical inguinal orchidectomy performed between 1983 and 1988. Their age range was 22–74 years (median 36 years). They have been followed for a median of 5 years and 2 months from orchidectomy (range 14 months to 141 months).

Initial staging investigations prior to registering the patient for surveillance always included thorough physical examination, assay of serum concentrations of the beta sub-unit of human chorionic gonadotrophin (HCG) and alpha-fetoprotein (AFP), CT scan of thorax abdomen and pelvis, lymphogram and chest X-ray. The histopathology had always been reviewed in the Department of Histopathology of The Royal Marsden Hospital and the diagnosis of pure seminoma confirmed. The method of surveillance is illustrated in Figure 1 and it can be seen that outpatient clinic assessments were performed every 2 months during the first year after orchidectomy, every 3 months during the second year, and every 4 months during the third year and thereafter less often. During the first year anteroposterior and oblique abdominal X-rays were performed to follow retroperitoneal lymph nodes, but lymphangiography was not repeated once the quantity of contrast had diminished. Abdominal CT scans were performed annually, or earlier if the patient had symptoms suggestive of recurrence. Re-staging after diagnosis of relapse always included a CT scan of the thorax abdomen and pelvis, and repeat assays of serum HCG and AFP.

The histological review of the primary tumours was performed by two pathologists (NA and CF). Sections were examined for the following features:

(1) The pattern of seminoma (classical, fibrous bands, angiomatoid).
(2) Syncytiotrophoblastic giant cells, necrosis, stromal granulomas, lymphoid infiltrate, and Leydig cells (all scored semiquantitatively).
(3) The cellular content of seminiferous tubules in adjacent residual testis, assessed as: no cells; Sertoli cells only; spermatogenic activity; *in situ* germ cell neoplasia (extent recorded semiquantitatively).
(4) Invasion of spermatic cord, rete testis and vessels (Blood or lymphatic). Lymphatic and vascular invasion were sometimes difficult to distinguish and in these cases invasion was deemed to be of lymphatics unless red blood cells were also seen within the vessel.

Patients who relapsed with small volume disease $<5$ cm in diameter in the para-aortic nodes were then treated with radiotherapy, using a 'dogleg' field encompassing para-aortic and ipsilateral pelvic lymph nodes. Treatment was with 6–8 MEV linear accelerators trating anterior and posterior portals daily to a midplane dose in the para-aortic region of 35 Gy in 18 fractions over 3½ weeks restricting the dose to

Correspondence: A. Horwich, Academic Unit Department of Radiotherapy and Oncology, The Royal Marsden Hospital, Downs Road, Sutton, Surrey SM2 5PT, UK.
Supported by grants from the Cancer Research Campaign and The Bob Champion Cancer Trust and by Research Funds at The Royal Marsden Hospital.
Received 16 October 1991; and in revised form 20 December 1991.

| | *Seminoma stage I surveillance* | | | | | | | | | | | | | | | |
|---|---|---|---|---|---|---|---|---|---|---|---|---|---|---|---|---|
| Month | 1 | 2 | 3 | 4 | 5 | 6 | 7 | 8 | 9 | 10 | 11 | 12 | 18 | 24 | 30 | 36 |
| OPD | + | + | + | + | + | + | + | + | + | + | + | } | Then as below | | | |
| Markers | + | + | + | + | + | + | + | + | + | + | + | } | | | | |
| CXR | + | + | + | + | + | + | + | + | + | + | + | } | | | | |
| Abd.XR | + | + | + | + | + | + | + | + | + | + | + | } | | | | |
| CT Scan | | | | | | | | | | | + | | | + | | + |
| U/S Abd. | | | | | | | | | | | | | + | | + | |
| | Year 2 | | OPD q 2/12 | | | U/S @ 18/12 | | | CT @ 24/12 | | | | | | | |
| | Year 3 | | OPD q 3/12 | | | U/S @ 30/12 | | | CT @ 36/12 | | | | | | | |
| | Year 4 | | OPD q 4/12 | | | | | | | | | | | | | |
| | Year 5 | | OPD q 6/12 | | | | | | | CT @ 60/12 | | | | | | |

OPD = Outpatient visit. CXR = Chest X-ray. Abd. X-ray = Plain abdominal films to follow lymphogram. CT = Computer tomographic. U/S = Ultrasound.

**Figure 1** The Royal Marsden Hospital surveillance protocol for stage I testicular seminoma. OPD = Outpatient visit. CXR = Chest X-ray. Abd. X-ray = Plain abdominal films to follow lymphogram. CT = Computer tomographic. U/S = Ultrasound.

the pelvis to 30 Gy in 15 fractions over 3 weeks. Patients with large volume abdominal relapse, disseminated relapse, or second relapse were treated with chemotherapy using single agent carboplatin (Horwich *et al.*, 1989), and then with radiotherapy if the recurrence was localised. The radiation technique was as described above with target volume restricted to the post-chemotherapy mass and adjacent lymph nodes.

### Statistics

Relapse free survival and survival were measured from the date of registration. One patient developed a second primary tumour of the head and neck. His seminoma had previously recurred, however, for analysis of cause specific survival, he was censored at the date of the second tumour. Differences between relapse free survival curves in relation to histopathological risk factors were analysed by the logrank method.

### Results

In 103 patients fully evaluated for histological analysis and managed by surveillance for a median of 62 months after orchidectomy for stage I seminoma, 17 have relapsed and the probability of remaining relapse free at 5 years was 82% (95% confidence interval, 74%–88%) (Figure 2). No patients have died of germ cell tumour; three died of coincidental disease. The site and extent of disease at relapse is shown in Table I. Of the 17 patients recurrence was discerned with small volume retroperitoneal nodes <5 cm in diameter in 14. One patient did not have a recurrence detected until his para-aortic lymph node was 6 cm in diameter. Another patient had para-aortic nodes more than 5 cm in diameter associated with mass in the inguinal region at the upper part of the ipsilateral inguinal canal, and a further patient relapsed with multiple para-aortic lymph nodes 2–5 cm in diameter associated with a solitary lung nodule assumed to represent a metastasis. The nodule was no longer detectable on post-chemotherapy scans.

Of the six patients relapsing with nodes <2 cm in diameter, one had previously had radiotherapy because of a contralateral testicular tumour diagnosed 11 years previously. The other five were treated with radiotherapy. Of the eight patients relapsing with nodes between 2 and 5 cm in diameter, seven were treated with radiotherapy, but one patient with a 5 cm mass obstructing the ureter was treated initially with chemotherapy and then with radiotherapy. Thus 12 of the 17 relapsing patients were treated with radiotherapy alone, the remaining five had chemotherapy alone (three) or chemotherapy plus radiotherapy (two).

Of the 12 patients initially treated with radiotherapy for recurrence, four developed a second recurrence in mediastinal (three) or supraclavicular (one) nodes at 5, 6, 10 and 19 months after retroperitoneal node irradiation. All these four were subsequently treated with carboplatin chemotherapy and in three cases the site of relapse was treated with radiotherapy also. Thus of the 17 patients relapsing from surveillance, eight required chemotherapy at some stage because of the extent of relapse and one required chemotherapy because of prior irradiation.

The analysis of the influence of histological parameters on risk of relapse did not reveal any significant factors except for lymphatic and vascular invasion (Figure 3). Four of 42 patients with neither lymphatic nor vascular invasion recur-

**Table I** Stage I seminoma: pattern of relapse on surveillance

| *Relapse pattern* | *Number of patients* | *Treatment of relapse* |
|---|---|---|
| P.A. nodes <2 cm | 6 | RT-5<br>CT-1[a] |
| P.A. nodes 2–5 cm | 8 | RT-7<br>CT + RT-1[b] |
| P.A. nodes >5 cm | 1 | CT + RT |
| P.A. nodes >5 cm and inguinal nodes | 1 | CT |
| P.A. nodes 2–5 cm and lung | 1 | CT |

[a]Prior abdominal radiotherapy. [b]5 cm mass invading ureter. RT = Radiotherapy. CT = Chemotherapy.

**Figure 2** Surveillance protocol for stage I testicular seminoma; relapse-free survival in the 103 patients evaluable for analysis of histopathological risk factors.

**Figure 3** Relapse-free survival with respect to microscopic invasion of blood vessels and/or lymphatic vessels; 1 = neither present, 2 = one of either blood vessel or lymphatic invasion, 3 = both blood vessel and lymphatic invasion.

**Figure 4** Relapse-free survival with respect to microscopic invasion of lymphatic vessels; 1 = invasion, 2 = no invasion.

**Figure 5** Relapse-free survival with respect to microscopic invasion of blood vessels; 1 = invasion; 2 = no invasion.

red, nine of 53 patients with either lymphatic or vascular invasion recurred and three of eight cases with both lymphatic and vascular invasion recurred (*P* = 0.05-trend). The individual analyses of lymphatic invasion (Figure 4) and of vascular invasion (Figure 5) showed that these conferred a slightly higher relapse risk which did not reach statistical significance.

### Discussion

This report on surveillance for stage I seminoma of the testis brings up-to-date our previous report (Duchesne *et al.*, 1990). The recurrence rate is slightly higher with longer follow-up with an actuarial risk of recurrence of 18% by 5 years. The predominant pattern of relapse was within 3 years of orchidectomy and within para-aortic lymph nodes. The latest relapse of our series was at 4 years post-orchidectomy and the median follow-up of the 103 patients was just over 5 years.

A report on surveillance for stage I seminoma from the Princess Margaret Hospital, Toronto (Thomas *et al.*, 1989) was based on 81 patients followed for a median of 19 months. At that time only three patients had relapsed at 3, 5 and 18 months after orchidectomy, however, with longer follow-up, a higher recurrence rate has been observed close to that noted in this report (G. Thomas, personal communication). Similarly a Danish Study of surveillance for stage I seminoma has found a very similar relapse rate (Specht *et al.*, 1991). It therefore, seems likely that even with long follow-up less than 20% of patients with clinical stage I seminoma will relapse and the benefit of surveillance is to spare more than 80% of patients the possible side-effects of adjuvant radiotherapy including peptic ulceration (Hamilton *et al.*, 1987) and induction of second malignancies (Hay *et al.*, 1984; Smith & Doll, 1982). On the other hand, radiotherapy is usually very well tolerated; peptic ulcer occurs very predominantly in patients with a prior history of either ulceration or abdominal surgery (Hamilton *et al.*, 1987) and the risk of radiation induced second tumour appears extremely small (Fossa *et al.*, 1990). Furthermore, this report illustrates firstly the difficulty of identifying any subgroup of patients with a particularly high or low risk of relapse on surveillance and also the problems of carrying out the policy safely since four of the 17 relapsing patients had moderately bulky disease at relapse. It is therefore considered that at present surveillance should remain a research protocol and the routine management of the patient with stage I seminoma should be with adjuvant retroperitoneal irradiation post-orchidectomy. The pattern of recurrence in para-aortic lymph nodes would suggest that the majority of patients may not benefit from the pelvic component of the standard 'dogleg' field, though this conclusion would not apply to those with a history of prior inguinal surgery (Mason *et al.*, 1991); a current Medical Research Council trial in stage I testicular seminoma is prospectively comparing 'dogleg' with para-aortic field radiotherapy following orchidectomy.

### References

CULLEN, M. (1991). Management of stage I non-seminoma: surveillance and chemotherapy. In Horwich, A. (ed.). *Testicular Cancer – Clinical Investigation and Management*. London, New York, Tokyo, Melbourne, Madras: Chapman and Hall Medical, 269–288.

DUCHESNE, G.M., HORWICH, A. & DEARNALEY, D.P. (1990). Orchidectomy alone for stage I seminoma of the testis. *Cancer*, **65**, 1115–1118.

FOSSA, S.D., LANGMARK, N., AASS, A., ANDERSEN, R., LOTHE, R. & BORRESEN, A.L. (1990). Second non-germ cel malignancies after radiotherapy of testicular cancer with or without chemotherapy. *Br. J. Cancer*, **61**, 639–643.

FREEDMAN, L.S., PARKINSON, M.C., JONES, W.G., OLIVER, R.T.D., PECKHAM, M.J., READ, G., NEWLANDS, E.S. & WILLIAMS, C.J. (1987). Histopathology in the prediction of relapse of patients with stage I testicular teratoma treated by orchidectomy alone. *Lancet*, **ii**, 294–298.

HAMILTON, C.R., HORWICH, A., BLISS, J.M. & PECKHAM, M.J. (1987). Gastrointestinal morbidity of adjuvant radiotherapy in stage I malignant teratoma of the testis. *Radioth. & Oncol.*, **10**, 85–90.

HAMILTON, C.R., HORWICH, A., EASTON, D. & PECKHAM, M.J. (1986). Radiotherapy for stage I seminoma testis: results of treatment and complications. *Radioth. & Oncol.*, **6**, 115–120.

HAY, J.H., DUNCAN, W. & KERR, G.R. (1984). Subsequent malignancies in patients irradiated for testicular tumours. *Br. J. Radiol.*, **57**, 597–602.

HORWICH, A., DEARNALEY, D.P., DUCHESNE, G.M., WILLIAMS, M., BRADA, M. & PECKHAM, M.J. (1989). Simple non-toxic treatment of advanced metastatic seminoma with carboplatin. *J. Clin. Oncol.*, **7**, 1150–1156.

HORWICH, A. & PECKHAM, M.J. (1988). Surveillance after orchidectomy for clinical stage I germ cell tumours of the testis. In: Schröder, F.H., Klijn, J.G.M., Kurth, K.H., Pinedo, J.M., Splinter, T.A.W. & De Voogt, H.J. (eds). *Progress and Controversies in Oncological Urology II*, Vol 269. New York: Alan R. Liss Inc., 471–478.

MAIER, J.G., SULAK, M.H. & MITTEMEYER, B.T. (1968). Seminoma of the testis: analysis of treatment success and failure. *Am. J. Roentgenol.*, **102**, 596–602.

MASON, M.D., FEATHERSTONE, J., OLLIFF, J. & HORWICH, A. (1991). Inguinal iliac lymph node involvement in germ cell tumours of the testis: implications of radiological investigation and for therapy. *Clin. Oncol.*, **3**, 147–150.

SMITH, P.G. & DOLL, R. (1982). Mortality among patients with ankylosing spondylitis after a single treatment course with X-rays. *Br. Med. J.*, **284**, 449–460.

SPECHT, L., VON DER MAASE, H., JACOBSEN, G.K. & THE DATECA STUDY GROUP (1991). Prognostic factors for relapse in seminoma stage I treated with orchidectomy alone. (Abstract). *Eur. J. Cancer*, Supp 2, 108.

THOMAS, G.M., STURGEON, J.F., ALISON, M., JEWETT, M., GOLDBERG, S., SUGAR, L., RIDEOUT, D., GOSPODAROWICZ, M.K. & DUNCAN, W. (1989). A study of post-orchidectomy surveillance in stage I testicular seminoma. *J. Urol.*, **142**, 313–316.

ZAGARS. G.K. (1991). Management of stage I seminoma: radiotherapy. In Horwich, A. (ed.). *Testicular Cancer – Clinical Investigation and Management*. London, New York, Tokyo, Melbourne, Madras: Chapman and Hall Medical, 146–196.